# TRAF6 controls T cell homeostasis by maintaining the equilibrium of MALT1 scaffolding and protease functions

**DOI:** 10.3389/fimmu.2023.1111398

**Published:** 2023-01-24

**Authors:** Thomas J. O’Neill, Andreas Gewies, Thomas Seeholzer, Daniel Krappmann

**Affiliations:** Research Unit Signaling and Translation, Group Signaling and Immunity, Molecular Targets and Therapeutic Center, Helmholtz Center Munich, German Research Center for Environmental Health, Munich, Germany

**Keywords:** T cell signaling, CBM signalosome, autoimmunity, inflammation, NF-kappaB, MALT1 paracaspase, ubiquitin ligase, TRAF6

## Abstract

MALT1 is a core component of the CARD11-BCL10-MALT1 (CBM) signalosome, in which it acts as a scaffold and a protease to bridge T cell receptor (TCR) ligation to immune activation. As a scaffold, MALT1 binds to TRAF6, and T cell-specific TRAF6 ablation or destruction of MALT1-TRAF6 interaction provokes activation of conventional T (Tconv) effector cells. In contrast, MALT1 protease activity controls the development and suppressive function of regulatory T (Treg) cells in a T cell-intrinsic manner. Thus, complete loss of TRAF6 or selective inactivation of MALT1 catalytic function in mice skews the immune system towards autoimmune inflammation, but distinct mechanisms are responsible for these immune disorders. Here we demonstrate that TRAF6 deletion or MALT1 paracaspase inactivation are highly interdependent in causing the distinct immune pathologies. We crossed mice with T cell-specific TRAF6 ablation (*Traf6*-ΔT) and mice with a mutation rendering the MALT1 paracaspase dead in T cells (*Malt1* PD-T) to yield *Traf6*-ΔT;*Malt1* PD-T double mutant mice. These mice reveal that the autoimmune inflammation caused by TRAF6-ablation relies strictly on the function of the MALT1 protease to drive the activation of Tconv cells. Vice versa, despite the complete loss of Treg cells in *Traf6*-ΔT;*Malt1* PD-T double mutant mice, inactivation of the MALT1 protease is unable to cause autoinflammation, because the Tconv effector cells are not activated in the absence of TRAF6. Consequentially, combined MALT1 paracaspase inactivation and TRAF6 deficiency in T cells mirrors the immunodeficiency seen upon T cell-specific MALT1 ablation.

## Introduction

Antigen ligation to the T cell receptor (TCR) leads to signal transduction and NF-κB transcription factor activation, required for cellular survival, proliferation, differentiation and function of T lymphocytes. NF-κB signaling therefore constitutes an initial step in activation of the adaptive immune response, and mutations affecting NF-κB have wide-reaching consequences for T cell activation and homeostasis, ranging from immune suppression to autoimmune activation (1, 2). Following TCR antigen ligation, protein kinase signaling leads to assembly of the CBM complex, composed of caspase recruitment domain family member 11 (CARD11), B-cell lymphoma/leukemia 10 (BCL10), and mucosa-associated lymphoid tissue 1 (MALT1). Within the CBM complex, MALT1 plays an intriguing dual role as both a scaffold, acting as a binding platform for the E3 ligase tumor necrosis factor (TNF) receptor-associated factor 6 (TRAF6), and as a paracaspase, cleaving substrates with roles in T cell signaling, transcription and RNA stability (3).

Mouse models have revealed that MALT1 and TRAF6 play central roles in balancing immune activation and homeostasis. *Malt1* KO mice have simultaneous severe defects in conventional T (Tconv) and regulatory T (Treg) cells, resulting in a net outcome of immunodeficiency (4, 5). *Malt1* paracaspase dead (PD) mice, carrying a C472A exchange in the catalytic center, display only partially impaired Tconv effector cell responses, but strong defects in Treg cell numbers and functions, triggering an immune imbalance that leads to autoimmune inflammation (5–10). The essential role of TRAF6 and its interaction with MALT1 in TCR-dependent NF-κB signaling has been demonstrated *in vitro* (11–14). TRAF6 deficiency causes embryonal or perinatal lethality in mice (15), but mice with conditional *Traf6* KO in T cells (*Traf6*-ΔT) are viable (16). *Traf6*-ΔT mice suffer from autoimmunity associated with enhanced activation of Tconv effector cells that are unresponsive to the suppression of Treg cells. Importantly, *Malt1* TBM-T (TRAF6-binding mutant in T cells) mice with conditional destructive missense mutations in T cells rendering TRAF6 incapable of interacting with MALT1 show a highly similar autoimmune phenotype to *Traf6*-ΔT mice, demonstrating that the interaction of MALT1 and TRAF6 in T cells is critical for maintaining immune homeostasis (17). While loss of TRAF6 or MALT1-TRAF6 interaction abrogates TCR-induced NF-κB activation, the MALT1 protease is constitutively activated, leading to continuous substrate cleavage even in resting T cells (17).

While the fatal autoinflammation caused by the destruction of MALT1-TRAF6 binding in all cells is rescued by genetic inactivation of MALT1 paracaspase function, it remained unclear, if the T cell activation and autoimmunity caused by complete absence of TRAF6 in T cells is also driven by MALT1 protease activation. Treatment of *Traf6*-ΔT mice with a potent MALT1 inhibitor ameliorated some disease symptoms (17), but it remained elusive whether only T cells or also other cells are targeted in such a pharmacological setting. Thus, in order to provide evidence that loss of TRAF6 induces autoimmunity through cell-intrinsic MALT1 protease activation, we generated mice in which TRAF6 deletion and MALT1 protease inactivation are combined specifically in T cells. We demonstrate that MALT1 protease activity drives T cell activation upon loss of TRAF6. Vice versa, TRAF6 is essential for autoimmunity upon MALT1 protease inactivation in T cells, emphasizing the critical interdependency of MALT1 and TRAF6 to balance T cell activation and homeostasis.

## Materials and methods

### Mice

All mouse experiments were performed in accordance with the guidelines of the Federation of European Laboratory Animal Science Association and were approved by the Regierung von Oberbayern (ref. no. 55.2-2532-VET_02-17-122).

*Malt1* and *Traf6* floxed (fl) mice were derived from the European Conditional Mouse Mutagenesis (EUCOMM) program with generation described in (17). *Malt1* ^fl/fl^ and *Traf6* ^fl/fl^ mice were crossed to generate double-floxed *Malt1* ^fl/fl^;*Traf6* ^fl/fl^ mice. *Malt1* PD mice were provided by Rudi Beyaert (VIB, Ghent, Belgium) and generated as described (18, 19). Double floxed mice were crossed with *Malt1* ^PD/+^;*Traf6* ^wt/fl^;*CD4-Cre* ^+^ to generate *Traf6*-ΔT;*Malt1* PD-T (*Traf6* ^fl/fl^;*Malt1* ^PD/fl^;*CD4-Cre* ^+^) and *Wt* ^het^ (*Traf6* ^fl/+^;*Malt1* ^fl/+^;*CD4-Cre* ^+^), *Traf6*-ΔT (*Traf6* ^fl/fl^;*Malt1* ^fl/+^;*CD4-Cre* ^+^) and *Malt1* PD-T(*Traf6* ^fl/+^;*Malt1* ^PD/fl^;*CD4-Cre* ^+^) control groups.

### Flow cytometry

Lymphocyte populations were analyzed from peripheral (spleen and lymph nodes) and central (thymus) lymphoid organs. Tissue was meshed through a 100 µm strainer and treated with red blood cell lysis buffer (Miltenyi, 130-094-183). One million cells per staining were transferred to a 96-well plate, washed twice with cold phosphate-buffered saline (PBS) (350g, 5 min, 4°C) and stained with eFluor780 Live/Dead dye (eBioscience, 65-0865-18; 1:1000 in PBS, 30 min, 4°C). Cells were washed once with FCM buffer (3% fetal bovine serum in PBS) and treated with anti-CD16/CD32 Fc-block (eBioscience, 14-0161-85; 1:200 in FCM buffer, 20 min, RT). Supernatant was removed, and cells were stained with surface antibodies in FCM buffer. Staining was performed with anti-CD3-PECy7 (1:300, 25-0031-82, RRID: AB_469572), anti-CD45R-PerCP (1:200, Biolegend, 103234, AB_893353), anti-CD8a-FITC (1:100, 11-0081-85, RRID: AB_464916), anti-CD4-PE (1:300, 12-0042-85, RRID: AB_465512), anti-CD4-PerCP-Cy5.5 (1:300, 45-0042-82, RRID: AB_1107001), anti-CD44-PECy7 (1:400, 25-0441-82, RRID: AB_469623), anti-CD62L-APC (1:300, BD Pharmingen, 553152, RRID : AB_398533), anti-CD69-APC (1:200, 17-0691-82, RRID: AB_1210795), and anti-ICOS-FITC (1:200, 11-9949-82, RRID: AB_465458). For intracellular staining of FoxP3, cells were fixed and permeabilized using the FoxP3/transcription factor staining buffer set (eBioscience, 00-5523-00; 1 h, RT), washed with permeabilization buffer (eBiosciences, 00-8333-56) and stained with anti-FoxP3-PE (12-5773-82, RRID: AB_465936) in permeabilization buffer. Cells were washed with permeabilization buffer, resuspended in FCM buffer, and measured using an Attune Acoustic Focusing Cytometer (Thermo Fisher). All antibodies are from eBiosciences except where indicated. Gating strategies for detecting the different cell subsets are shown in **Figure S1**.

### Cytokine and autoantibody analysis

The cytokine TNFα and anti-double-stranded (ds)DNA autoantibodies were measured in mouse serum *via* flow cytometry using a cytometric bead array kit (562246 and 562336, BD) and anti-dsDNA Ig’s kit (Catalog #5110, Alpha Diagnostic International) according to manufacturer’s recommendations.

### Stimulation and biochemical analyses of purified CD4 T cells

Primary murine splenocytes were isolated from spleen and treated with Red Blood Cell Lysis Solution (Miltenyi) and CD4^+^ T cells were purified using CD4^+^ T cell isolation kit II (Miltenyi) according to the manufacturer’s protocol. CD4^+^ T cells were cultured in primary T cell medium (RPMI 1640, 100 U/ml penicillin, 100 µg/ml streptomycin, 10% heat inactivated fetal calf serum, 10 mM HEPES pH 7.5, 2 mM L-Glutamine, 1 mM Sodium-Pyruvate, MEM-NEAA (1x), 50 nM ß-Mercaptoethanol [ll Gibco]). For stimulation, cells were treated with Phorbol 12-Myristate 13-Acetate (PMA (P), 200 ng/ml; Merck)/Ionomycin (Iono (I), 300 ng/ml; Calbiochem) for 30 min. For Western blotting, cells were lysed in co-immunoprecipitation (co-IP) buffer (25 mM HEPES pH 7.5, 150 mM NaCl, 0.2% NP-40, 10% glycerol, 1 mM DTT, 10 mM NaF, 8 mM ß-glycerophosphate, 300 µM sodium vanadate and protease inhibitor cocktail mix (Roche)) for 20 min at 4°C. Cellular lysis for electrophoretic mobility shift assay (EMSA) samples was performed in high salt buffer (20 mM HEPES pH 7.9, 350 mM NaCl, 20% glycerol, 1 mM MgCl_2_, 0.5 mM EDTA, 0.1 mM EGTA, 1% NP-40, 1 mM DTT, 10 mM sodium fluoride, 8 mM β-glycerophosphate, 300 µM sodium vanadate and Roche protease inhibitor cocktail mix). Western blotting and EMSA were performed as previously described (20). Western blot antibodies: anti-ß-Actin (C4, 1:20.000; #sc-47778; RRID: AB_2714189), anti-CYLD (E-10; #sc-74435; RRID: AB_1122022), anti-HOIL-1 (H-1; #sc-393754; RRID: N/A), (all Santa Cruz); anti-Regnase-1 (#MAB7875; RRID: N/A) (R&D); HRP-conjugated anti-rabbit (#711-035-152; RRID: AB_10015282), HRP-conjugated anti-mouse (#715-035-150; RRID: AB_2340770), (all Jackson ImmunoResearch, 1:7000); all antibodies were used at 1:1000 dilution.

## Results

**Figure 1:**
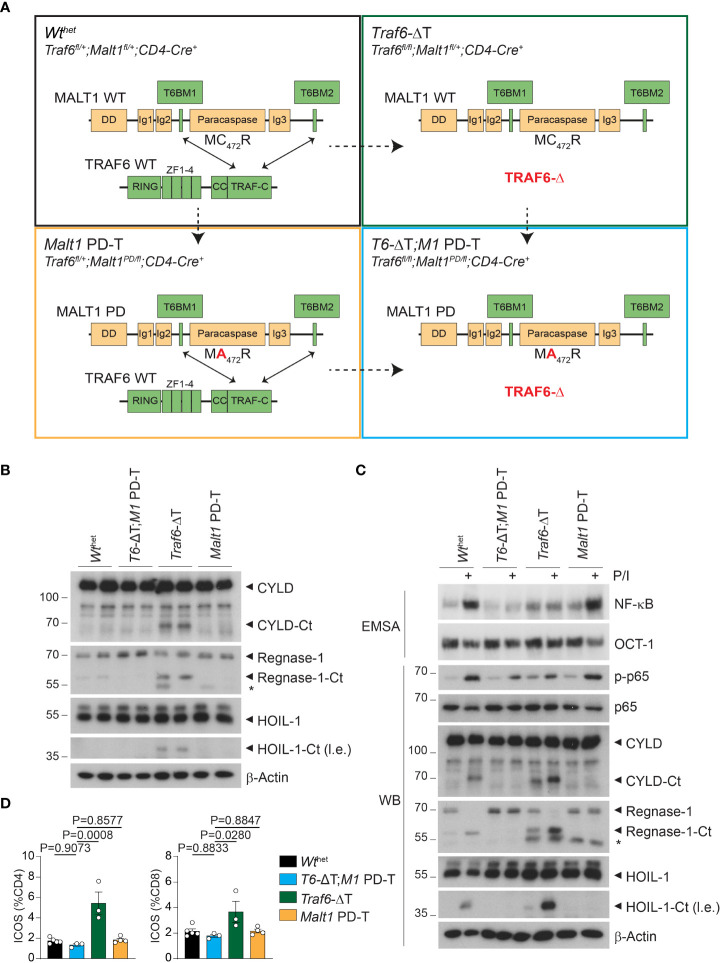
Effects of single or combined TRAF6 KO and MALT1 paracaspase mutation on signaling in CD4^+^ T cells. **(A)** Schematic overview of the four conditional mouse strains used in the analyses. **(B)** Western blots showing MALT1 substrate cleavage in unstimulated purified CD4^+^ T cells from *Wt* ^het^, *T6-*ΔT;*M1* PD-T, *Traf6*-ΔT and *Malt1* PD-T mice (two independent mice each). Asterisks indicate unspecific signals. **(C)** Analyses of NF-κB activation (EMSA), p65 phosphorylation and MALT1 substrate cleavage (Western blot, WB) in PMA/Ionomycin (P/I) stimulated purified CD4^+^ T cells of mice as depicted in **(A)**. **(D)** Expression of ICOS on CD4^+^ and CD8^+^ T cells by flow cytometric analysis of spleen of mice as depicted in **(A).** Bars show the means ± SEM, and P values were calculated by one-way ANOVA with Tukey’s multiple comparison test. All analyses were performed with mice 9-11 weeks of age. Each dot represents one mouse. Ct, C-terminus; l.e., long exposure.

The genetic disruption of MALT1 protease activity in *Malt1* PD-T mice causes autoimmunity in a T cell intrinsic manner (18). Further, T cell-specific deletion of TRAF6 in *Traf6*-ΔT mice or loss of MALT1-TRAF6 binding in *Malt1* TBM mice induces autoimmune inflammation (16, 17). To better understand how the interplay between MALT1 protease activity and TRAF6 balances T cell activation and homeostasis, we combined TRAF6 deletion and expression of MALT1 PD specifically in T cells. For this, we crossed mice to yield homozygous *Traf6* ^fl/fl^ and heterozygous *Malt1* ^C472A/fl^ together with CD4-Cre, which inactivates the two *Traf6* and one *Malt1* Wt floxed alleles at the CD4/CD8 double positive stage of T cell differentiation. Thus, the resulting *Traf6-*ΔT;*Malt1* PD-T (*Traf6* ^fl/fl^;*Malt1* ^PD/fl^;*CD4-Cre* ^+^) mice expressed the MALT1 paracaspase dead (PD) mutant in the absence of TRAF6. The immune phenotype of *Traf6-*ΔT;*Malt1* PD-T mice was compared to double heterozygous ‘wildtype’ (*Wt* ^het^ *: Traf6* ^fl/+^;*Malt1* ^fl/+^;*CD4-Cre* ^+^), *Traf6*-ΔT (*Traf6* ^fl/fl^;*Malt1* ^fl/+^;*CD4-Cre* ^+^) and *Malt1* PD-T (*Traf6* ^fl/+^;*Malt1* ^PD/fl^;*CD4-Cre* ^+^) littermates (**Figure 1A**). All genotypes were born at approximate Mendelian ratios and showed no observable phenotypic changes upon birth. However, *Malt1* PM-T mice stopped thriving at approximately 10 weeks of age. As previously observed, *Malt1* PD-T mice showed hunched posture and developed ataxia (18). For animal welfare and best comparison, phenotypic analyses of all mice were performed at 9-11 weeks of age. The four genotypic groups did not differ substantially in body weight, spleen weight or total splenocytes (**Figures S2A–C**). Relative numbers of B and T lymphocytes were unchanged except for minor reductions in CD3^+^ and CD8^+^ T cells in *Traf6*-ΔT mice and CD4^+^ T cells in *Malt1* PD-T mice, which were all reverted to normal in the *Traf6*-ΔT;*Malt1* PD-T (*T6-*ΔT;*M1* PD-T) mice (**Figures S2D, E**).

We purified CD4^+^ T cells from two independent mice of all four cohorts to compare the effects of the single and combined mutations in biochemical assays. As previously observed, *Traf6*-ΔT mice display constitutive cleavage of the MALT1 substrates and NF-κB signaling regulators CYLD and HOIL-1, as well as the RNA-binding protein Regnase-1 in the absence of any *ex vivo* T cell stimulation (**Figure 1B**) (17). Constitutive substrate cleavage was prevented upon additional inactivation of MALT activity in *T6-*ΔT;*M1* PD-T mice, proving that it was directly caused by MALT1. Next, we examined MALT1 protease and NF-κB activation upon stimulation with PMA/Ionomycin (P/I), which mimics TCR/CD28 engagement. Cleavage of CYLD, HOIL-1 and Regnase-1 was further enhanced after P/I treatment of CD4^+^ T cells from *Traf6*-ΔT mice, while no constitutive or inducible cleavage was seen in T cells from *Malt1* PD-T or *T6-*ΔT;*M1* PD-T double mutant mice (**Figure 1C**). In sharp contrast, NF-κB activation monitored by gel shift assays and p-p65 levels by Western blotting was unaffected in T cells from *Malt1* PD-T mice, but TRAF6 deletion alone (*Traf6*-ΔT) or in combination with the protease dead MALT1 was unable to promote NF-κB activation (**Figure 1C**). Basal NF-κB activation was mildly increased in *Traf6*-ΔT or *Malt1* PD-T cells compared to *Wt* ^het^, which may be explained by the inflammatory environment from which these cells are derived (see below). Basal NF-κB levels appeared to be even further reduced in T cells of *T6-*ΔT;*M1* PD-T double mutant mice.

To determine functional effects of chronic MALT1 substrate cleavage, we monitored inducible T cell costimulator (ICOS) expression on T cells from the modified mice, because ICOS expression is repressed by the post-transcriptional regulators Regnase-1 and Roquin-1/2, both of which are inactivated by MALT1-catalyzed cleavage (21, 22). Indeed, numbers of CD4^+^ and CD8^+^ T cells with elevated ICOS levels were increased in *Traf6*-ΔT mice, whereas ICOS expression was reverted to normal levels in T cells from *T6-*ΔT;*M1* PD-T mice (**Figure 1D**). These results demonstrate that TRAF6 deficiency provokes chronic MALT1 paracaspase activity in a T cell-intrinsic manner, which leads to upregulation of targets that are under control of mRNA stability factors regulated by MALT1 protease.

**Figure 2:**
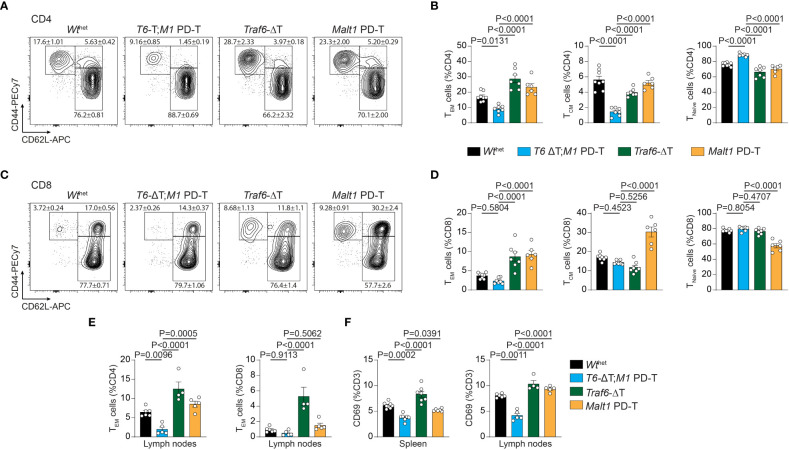
Effects of single or combined TRAF6 KO and MALT1 paracaspase mutation on T cell activation. **(A–D)** Flow cytometric analysis of CD44 and CD62L expression on CD4^+^ **(A, B)** and CD8^+^ **(C, D)** T cells with relative numbers of T_EM_ (CD44hi CD62Llo), T_CM_ (CD44hi CD62Lhi) and T_naïve_ (CD44lo CD62Lhi) cells in spleen of *Wt* ^het^, *T6-*ΔT;*M1* PD-T, *Traf6*-ΔT and *Malt1* PD-T mice. **(E)** Flow cytometric analyses of CD4^+^ and CD8^+^ CD44hi CD62Llo T_EM_ cells from lymph nodes of mice as depicted in **(A)**. **(F)** Relative numbers of CD3^+^ CD69^+^ T lymphocytes in spleen and lymph nodes of mice as depicted in **(A)**. Bars show the means ± SEM, and P values were calculated by one-way ANOVA with Tukey’s multiple comparison test. All analyses were performed with mice 9-11 weeks of age. Each dot represents one mouse.

Next, we determined the consequences of the various genetic alterations on the relative numbers of naïve T (T_naïve_), central memory T (T_CM_) and effector memory T (T_EM_) cells by measuring expression of CD44/CD62L on peripheral T cells (**Figures 2A–D** **;** **Figures S2F, G**). Both deletion of TRAF6 and inactivation of MALT1 paracaspase individually provoked an increase in numbers of splenic CD4^+^ T_EM_ cells, which coincided with a reduction in the T_naïve_ and T_CM_ cell populations (**Figures 2A, B**). This increase in the T_EM_ cell population was abolished and even decreased compared to *Wt* ^het^ in *T6-*ΔT;*M1* PD-T double mutant mice. Similar results were seen for frequencies of CD8^+^ T_EM_ cells (**Figures 2C, D**). However, while the increase in CD8^+^ T_EM_ cells in *Traf6*-ΔT mice primarily coincided with a decrease in T_CM_ cells, higher frequencies of CD8^+^ T_EM_ and T_CM_ populations in *Malt1* PD-T mice correlated with decreased T_naive_ cell numbers. Higher T_EM_ cell numbers were also detected in lymph nodes, especially in *Traf6*-ΔT mice, and the increase was abrogated in *T6-*ΔT;*M1* PD-T double mutant mice (**Figure 2E**). In line with increased T_EM_ populations, expression of the T cell activation marker CD69 was enhanced on CD3^+^ T cells in spleen and lymph nodes of *Traf6*-ΔT, and to a lesser extent on *Malt1* PD-T, and reduced on T cells from *T6-*ΔT;*M1* PD-T, even when compared to *Wt* ^het^ mice (**Figure 2F**). Therefore, combined TRAF6 deletion and MALT1 paracaspase inactivation in T cells reverts the cell-intrinsic T cell activation observed by single TRAF6 deficiency or MALT1 protease dead mutation.

**Figure 3:**
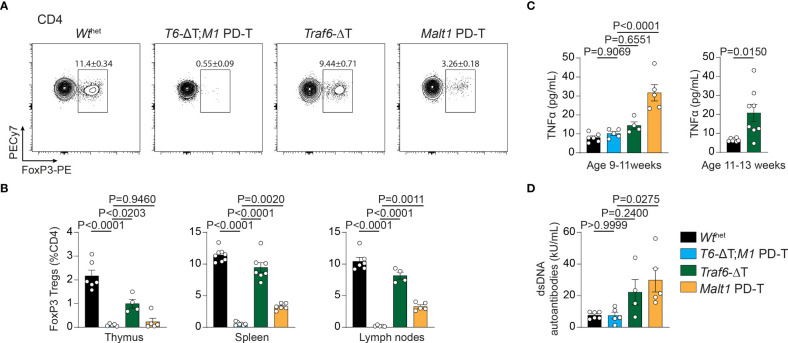
Effects of single or combined TRAF6 KO and MALT1 paracaspase mutation on Treg cell frequencies, TNFα and anti-dsDNA autoantibodies. **(A, B)** Flow cytometric analysis of splenic CD4^+^ FoxP3^+^ regulatory T (Treg) cells **(A)** with relative numbers of Treg cells from thymus, spleen and peripheral lymph nodes **(B)** of *Wt* ^het^, *T6-*ΔT;*M1* PD-T, *Traf6*-ΔT and *Malt1* PD-T mice. **(C)** Concentration of the cytokine TNFα in sera of mice as depicted in **(A)**. Right graph depicts TNFα concentrations in 11-13 week old *Traf6*-ΔT mice. **(D)** Concentrations of anti-dsDNA immunoglobulins in sera of mice as depicted in **(A)**. Bars show the means ± SEM, and P values were calculated by one-way ANOVA with Tukey’s multiple comparison test. All analyses were performed with mice 9-11 weeks of age except where otherwise stated. Each dot represents one mouse.

Developmental and functional defects in thymic and peripheral Treg cells are the underlying cause for the autoimmunity in MALT1 paracaspase defective mice (9, 23). In line with this, CD4^+^ FoxP3^+^ Treg cells in *Malt1* PD-T mice were severely reduced in spleen and lymph nodes and almost completely missing in the thymus (**Figures 3A, B**). In contrast, *Traf6*-ΔT mice displayed mildly reduced Treg cell frequencies in the thymus, while spleen and lymph nodes showed nearly normal numbers of Treg cells. However, combination of TRAF6 deletion and MALT1 protease inactivation in *T6-*ΔT;*M1* PD-T mice provoked a complete absence of Treg cells in the thymus and peripheral immune organs. Despite the severe reduction in thymic Treg cells, no genotype induced significant alterations in the frequency of double negative, double positive or single positive CD4+ or CD8+ T cells in the thymus, indicating that development of Tconv cells is not affected by TRAF6 deficiency and/or MALT1 protease inactivation (**Figures S3A–C**).

Finally, to determine the consequences of the single and combined mutations, we measured concentrations of the pro-inflammatory cytokine TNFα and anti-double-stranded DNA (dsDNA) antibodies in the sera of the mice as biomarkers for the onset of autoimmune inflammation. While TNFα and anti-dsDNA antibodies were upregulated in the serum of *Malt1* PD-T mice, there was only a tendency for an increase in *Traf6*-ΔT mice at 9-11 weeks (**Figures 3C, D**). However, the increase in TNFα was more pronounced at 11-13 weeks of age in *Traf6*-ΔT mice (**Figure 3D**). Further, we have shown that anti-dsDNA autoantibodies were elevated in older mice (17), indicating a slight delay in the onset of autoimmune inflammation in *Traf6*-ΔT compared to *Malt1* PD-T animals (**Figure 3D**). Upregulation of TNFα and anti-dsDNA autoantibody in the serum was abrogated in *T6-*ΔT;*M1* PD-T mice, revealing the interdependency of TRAF6 deletion and MALT1 paracaspase inactivation in triggering autoimmune inflammation.

## Discussion

**Figure 4:**
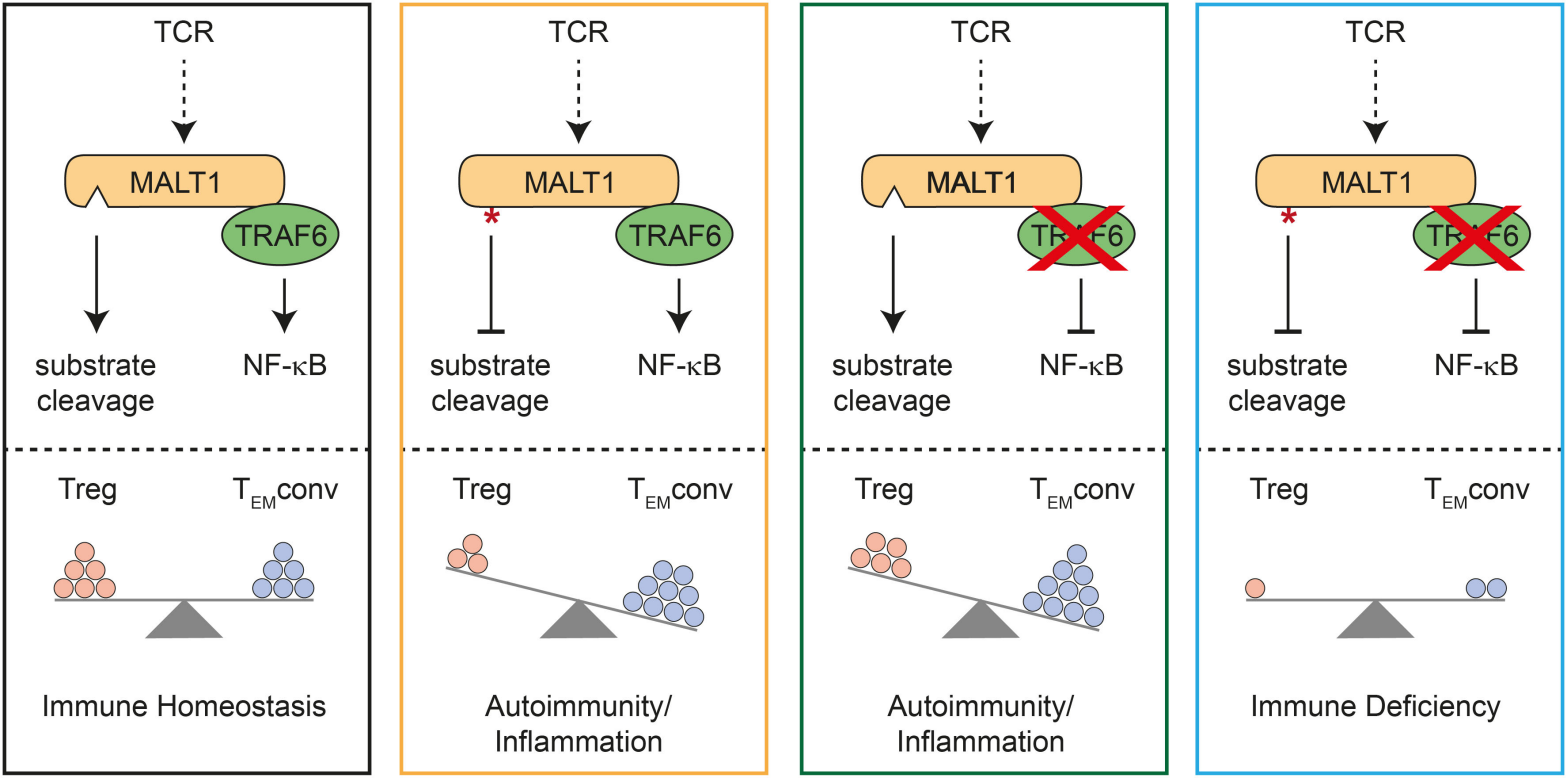
Schematic model for mutual control of homeostasis by MALT1 and TRAF6 in T cells. Effects of single or combined TRAF6 KO and MALT1 paracaspase mutation on NF-κB signaling and MALT1 substrate cleavage (upper part). Effects of single or combined mutations on regulatory T (Treg) and conventional T effector (T_EM_conv) cells, which determine the phenotypes of the mutant mice (lower part). Note that circles represent a combination of cell number and activation status in the different subsets.

By combining TRAF6 ablation and MALT1 paracaspase inactivation selectively in T cells, we provide genetic evidence that T effector responses and autoimmunity in the absence of TRAF6 relies on MALT1 protease activation (**Figure 4**). Vice versa, autoimmunity and inflammation triggered by MALT1 paracaspase inactivation is driven by TRAF6 and thus by activation of NF-κB signaling downstream of MALT1. In fact, TRAF6 and MALT1 paracaspase double mutations yield a reciprocal rescue of both autoimmune phenotypes, resulting in an immunodeficiency as described for global or T-cell specific MALT1-deficient mice (4, 5, 18). Thus, the fine-tuned equilibrium of MALT1 protease and scaffolding function determines the level of T cell activation and is critical for maintaining immune homeostasis.

Importantly, either destruction of MALT1 substrate cleavage or prevention of MALT1 downstream signaling by TRAF6 deletion in T cells provokes imbalanced immune signaling, which in both cases results in severe autoimmune inflammation (**Figure 4**). However, both immune pathologies are caused by deregulations in distinct T cell subsets. T or Treg cell-specific MALT1 inactivation leads to a ‘scurfy-like’ autoimmune syndrome, which is caused by impaired Treg cell development and function (9, 18). While induction of peripheral Treg cells especially in aged mice does not rely on MALT1, thymic Treg cells are lacking in the absence of either MALT1 or functional protease activation (6, 7, 18, 24). Of note, MALT1 was shown to regulate susceptibility of induced Treg cells to innate immune stimulation, and thus MALT1 has an indispensable function in balancing thymic versus peripheral tolerance (24). Importantly, MALT1 protease activity is required to maintain high expression of CTLA-4 on Treg cells and it was shown that even a moderate decrease in CTLA-4 expression can lead to autoimmunity (9, 18, 25). Thus, MALT1 protease is critical for maintaining peripheral immune tolerance, because *Malt1* PD Treg cells can no longer counteract the activation of Tconv effector cells (**Figure 4**). Loss of Treg cell control leads to autoimmune reactions in multiple tissues, even though conventional MALT1 paracaspase defective T cells are also functionally compromised in effector responses (6–8, 10, 18). In contrast, despite some decrease in thymic Treg cells, peripheral Treg cells are present in mice with conditional deletion of TRAF6 in T cells. In TRAF6-deficient mice, Treg cells are functional, but they are no longer able to counteract Tconv effector responses (16). Thus, loss of TRAF6 causes autoimmune inflammation primarily by enhancing conventional T effector cell responses, even in the presence of functional Treg cells (**Figure 4**). Both genetic perturbations cause T cell activation by distinct mechanisms, which may also explain other differences, such as the decrease or increase in CD8 T_CM_ cell numbers in *Traf6*-ΔT or *Malt1* PD-T mice, respectively. We previously showed that selective destruction of MALT1-TRAF6 interaction in T cells phenocopies the autoimmune inflammation induced be complete absence of TRAF6 in T cells (17). Further, symptoms of immune activation in *Traf6*-ΔT mice are ameliorated by systemic MALT1 protease inhibitor treatment. Here we demonstrate that ablation of TRAF6 in T cells induces autoimmunity *via* T cell-intrinsic activation of MALT1 substrate cleavage. Of note, TRAF6 is involved in many other innate immune and inflammatory signaling pathways (26), but the key roles of TRAF6 in triggering TCR-induced NF-κB signaling and protecting from uncontrolled T cell activation both rely upon its interaction with MALT1.

Treg cell development and function is not affected by destruction of MALT1-TRAF6 interaction and is only partially compromised by loss of TRAF6, despite strongly impaired TCR-induced NF-κB activation (16, 17). This was somewhat unexpected, because canonical NF-κB subunits p65 and c-Rel are critical in controlling Treg development and function (27, 28). Importantly, neither TRAF6 ablation nor lack of MALT1-TRAF6 interaction affects NF-κB activation in response to inflammatory TNFα (17), suggesting that other NF-κB inducers are able to compensate for the loss of TCR-induced NF-κB signaling. Thus, TCR stimulation seems to primarily drive Treg development and suppressor functions by providing the signal that induces MALT1 protease activation. It is worth mentioning that in older mice TRAF6 has a function in maintaining FOXP3 expression and thus Treg identity, which is independent of its interaction with MALT1 (17, 29, 30). Reminiscent to Treg cells, conventional T_EM_ cells develop in the absence of TRAF6 or MALT1-TRAF6 interaction and are thus also deprived of TCR-induced NF-κB. In this setting, chronic MALT1 protease activity seems to initiate T cell effector responses that drive an inflammatory milieu through the production of inflammatory cytokines such as TNFα, which in turn may act on T cells and compensate for the loss of TCR-triggered NF-κB activation (16, 17). Of note, this cell-intrinsic activation of conventional T cells causes autoimmune inflammation even in the presence of functional Treg cells (16, 17). Nevertheless, even though T cells lacking TRAF6 cause autoimmune inflammation, it is unclear in how far they would be able to mount a productive adaptive immune response upon infection. Clearly, only the combined mutation of MALT1 scaffolding and protease functions renders conventional T cells inactive, which results in immunodeficiency as observed in T cell-specific *Malt1* KO mice (**Figure 4**) (18). Thus, a tight balance of MALT1 signaling and proteolytic function in conventional and regulatory T cells is necessary for maintaining immune homeostasis and for allowing productive immune activation.

## Data availability statement

The original contributions presented in the study are included in the article/**Supplementary Material**. Further inquiries can be directed to the corresponding author.

## Ethics statement

All mouse experiments were performed in accordance with the guidelines of the Federation of European Laboratory Animal Science Association and were approved by the Regierung von Oberbayern (ref. no. 55.2-2532-VET_02-17-122).

## Author contributions

TO, AG and DK conceived the study and designed experiments. TO performed most immune phenotyping analyses in mice. TS conducted biochemical analyses in primary T cells. TO, AG, and DK wrote the manuscript. All authors contributed to the article and approved the submitted version.
