# Supplementary material for: TRAF6 controls T cell homeostasis by maintaining the equilibrium of MALT1 scaffolding and protease functions
Source: Front Immunol. 2023 Jan 24;14:1111398. doi: 10.3389/fimmu.2023.1111398 (PMC9902345; doi:10.3389/fimmu.2023.1111398)
Supplement: Supplementary file 1 [file DataSheet_1.pdf]

O'Neill et al

Supplementary Figures and Legends

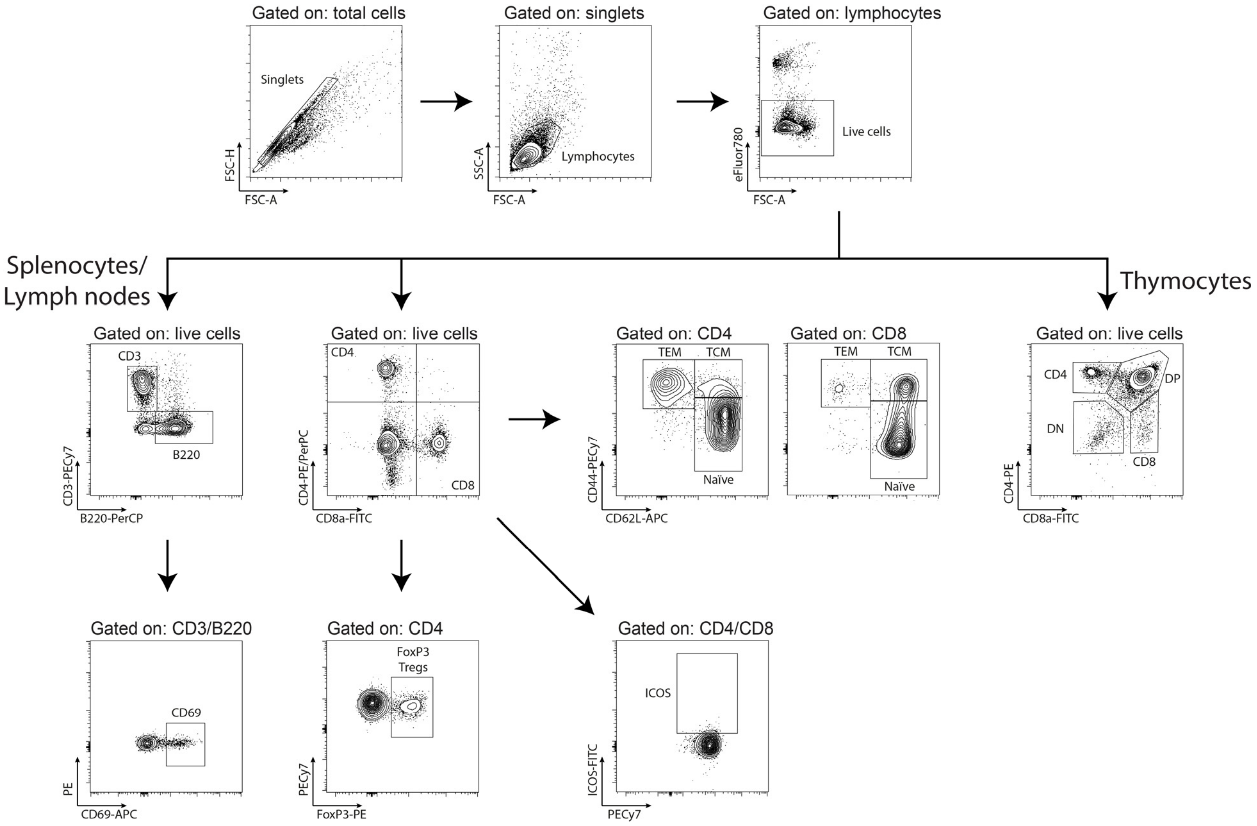

Figure S1: Gating strategies for flow cytometry analyses. All cell populations were gated on single lymphocytes based on forward and sideward scatter and on live cells based on the eFluor780 Live/Dead staining. Antibodies for stratification of T and B cell populations in spleen, lymph nodes and thymus are given in the Materials and Methods.

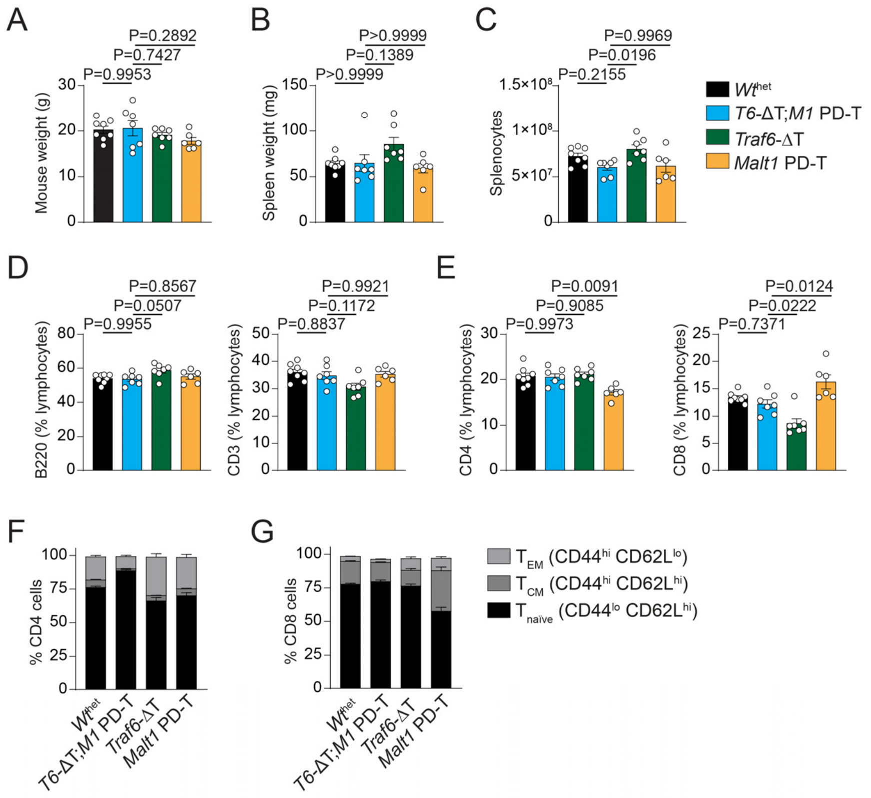

Figure S2: Phenotypic analyses of T cell populations. (A-C) Mouse weights (A), spleen weights (B) and total splenocyte numbers (C) of *Wt*^{het}, *T6*-ΔT;*M1* PD-T, *Traf6*-ΔT and *Malt1* PD-T mice. (D-E) Flow cytometric analysis of B220^{+} B cells and CD3^{+} T cells (D) and CD4^{+} and CD8^{+} T cells (E) from spleen of mice as depicted in A. (F-G) Flow cytometric analyses and relative numbers of T_{EM} (CD44hi CD62Llo), T_{CM} (CD44hi CD62Lhi) and $T_{naïve}$ (CD44lo CD62Lhi) CD4^{+} (F) and CD8^{+} (G) cells of *Wt*^{het}, *T6*-ΔT;*M1* PD-T, *Traf6*-ΔT and *Malt1* PD-T mice. Bars show the means ± SEM, and P values were calculated by one-way ANOVA with Tukey's multiple comparison test. All analyses were performed with mice 9-11 weeks of age. Each dot represents one mouse.

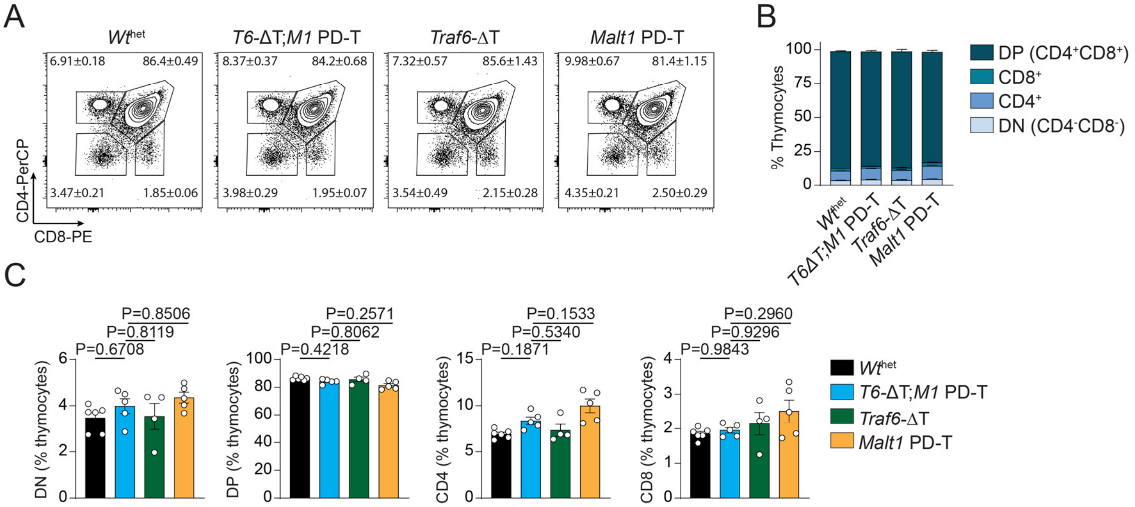

Figure S3: Thymic T cell development. (A to C) Flow cytometric analysis of thymic T cell populations (A) with relative numbers of double-negative, double-positive, and single-positive $CD4^+$ and $CD8^+$ T cells (B) and relative ratios of thymic T cell populations (C) of *Wt*^{het}, *T6*-ΔT;*M1* PD-T, *Traf6*-ΔT and *Malt1* PD-T mice. Bars show the means ± SEM, and P values were calculated by one-way ANOVA with Tukey's multiple comparison test. All analyses were performed with mice 9-11 weeks of age. Each dot represents one mouse.
